# Duration of SARS-CoV-2 RNA Shedding Is Significantly Influenced by Disease Severity, Bilateral Pulmonary Infiltrates, Antibiotic Treatment, and Diabetic Status: Consideration for Isolation Period

**DOI:** 10.3390/pathophysiology30020016

**Published:** 2023-05-04

**Authors:** Muhammad Vitanata Arfijanto, Tri Pudy Asmarawati, Bramantono Bramantono, Musofa Rusli, Brian Eka Rachman, Bagus Aulia Mahdi, Nasronudin Nasronudin, Usman Hadi

**Affiliations:** 1 Department of Internal Medicine, Faculty of Medicine, Airlangga University, Surabaya 60132, Indonesia; 2 Universitas Airlangga Hospital, Airlangga University, Surabaya 60115, Indonesia

**Keywords:** COVID-19, viral shedding, SARS-CoV-2, risk factors, infectious disease

## Abstract

Severe acute respiratory syndrome coronavirus type 2 (SARS-CoV-2) ribonucleic acid (RNA) shedding is an important parameter for determining the optimal length of isolation period required for coronavirus disease 2019 (COVID-19) patients. However, the clinical (i.e., patient and disease) characteristics that could influence this parameter have yet to be determined. In this study, we aim to explore the potential associations between several clinical features and the duration of SARS-CoV-2 RNA shedding in patients hospitalized with COVID-19. A retrospective cohort study involving 162 patients hospitalized for COVID-19 in a tertiary referral teaching hospital in Indonesia was performed from June to December 2021. Patients were grouped based on the mean duration of viral shedding and were compared based on several clinical characteristics (e.g., age, sex, comorbidities, COVID-19 symptoms, severity, and therapies). Subsequently, clinical factors potentially associated with the duration of SARS-CoV-2 RNA shedding were further assessed using multivariate logistic regression analysis. As a result, the mean duration of SARS-CoV-2 RNA shedding was found to be 13 ± 8.44 days. In patients with diabetes mellitus (without chronic complications) or hypertension, the duration of viral shedding was significantly prolonged (≥13 days; *p* = 0.001 and *p* = 0.029, respectively). Furthermore, patients with dyspnea displayed viral shedding for longer durations (*p* = 0.011). The multivariate logistic regression analysis reveals that independent risk factors associated with the duration of SARS-CoV-2 RNA shedding include disease severity (adjusted odds ratio [aOR] = 2.94; 95% CI = 1.36–6.44), bilateral lung infiltrates (aOR = 2.79; 95% CI = 1.14–6.84), diabetes mellitus (aOR = 2.17; 95% CI = 1.02–4.63), and antibiotic treatment (aOR = 3.66; 95% CI = 1.74–7.71). In summary, several clinical factors are linked with the duration of SARS-CoV-2 RNA shedding. Disease severity is positively associated with the duration of viral shedding, while bilateral lung infiltrates, diabetes mellitus, and antibiotic treatment are negatively linked with the duration of viral shedding. Overall, our findings suggest the need to consider different isolation period estimations for specific clinical characteristics of patients with COVID-19 that affect the duration of SARS-CoV-2 RNA shedding.

## 1. Introduction

Coronavirus disease 19 (COVID-19), an infectious disease caused by the novel severe acute respiratory syndrome coronavirus type-2 (SARS-CoV-2), encompasses varying manifestations ranging from asymptomatic and mild illness to life-threatening complications including acute respiratory distress syndrome (ARDS). The World Health Organization (WHO) announced COVID-19 as a pandemic on 11 March 2020; as of February 2023, over 758 million confirmed cases and over 6.8 million deaths had been reported [1]. In Indonesia, COVID-19 has affected over 6.7 million people and more than 160,000 deaths (2.4%) have been reported [2].

The duration of SARS-CoV-2 RNA shedding is the period from the onset of symptoms to the virus not being detected upon polymerase chain reaction (PCR) examination [3]. Some studies have reported that the viral shedding time of SARS-CoV-2 from infected patients is affected by transmission source, age, disease severity, drugs, comorbidities, and immunity status [3,4,5,6]. The most prolonged duration is about 60 days, and the shortest is about 3 days [7]. However, the duration of SARS-CoV-2 viral shedding is variable, and the pattern and influencing factors are yet to be determined.

Recognizing the prolonged viral shedding of SARS-CoV-2 is mandatory in deciding the isolation period and treatment of patients with COVID-19. The risk of transmission due to viral shedding cannot be ignored, even if there is a resolution that is not accompanied by any symptoms. The risks vary depending on the infectiousness level of the virus and the patient population involved [7].

The prolonged infection and evolution of the SARS-CoV-2 virus in immunocompromised individuals may lead to the emergence of new variants, the increase in virulence levels, and potential immune escape, thus posing the risk of complicating pandemic control [8,9]. Thus, clinical judgments should be made based on clinical features, guidelines, the detectable viral load, transmission source, infectiousness level, and patient-related factors [7,10,11,12,13,14]. It is critical to further study the factors affecting the viral shedding time of SARS-CoV-2 to estimate the optimal duration of isolation required by patients based on certain conditions. As such, we intend to investigate related factors, particularly host factors (age, sex, and comorbidities) and symptoms, with respect to the duration of SARS-CoV-2 viral shedding.

## 2. Materials and Methods

### 2.1. Study Design and Sample Selection

**Figure 1:**
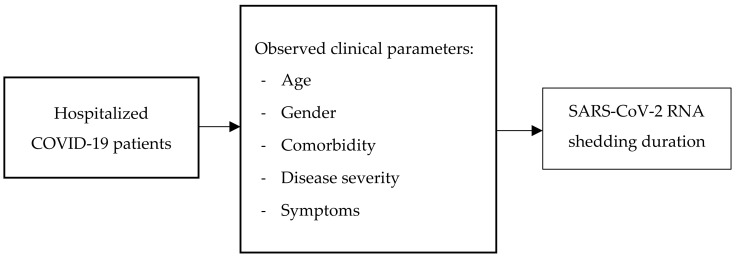
Retrospective observational study design to analyze the association between clinical parameters and the duration of SARS-CoV-2 RNA shedding.

This research is a single-center retrospective observational study on hospitalized COVID-19 patients in the isolation ward of a tertiary referral teaching hospital in Surabaya, Indonesia. This study was performed from June to December 2021. We aimed to analyze several factors, such as age, gender, comorbidity, disease severity, and COVID-19-related symptoms, and their association with the duration of SARS-CoV-2 RNA shedding in a retrospective design (Figure 1). The inclusion criteria were confirmed COVID-19 patients over 17 years old who had been hospitalized until being declared free from isolation after 2 consecutive negative RT-PCR tests. The exclusion criteria were confirmed COVID-19 patients who died or were discharged from the hospital before the RT-PCR test showed negative results. Diagnosis of COVID-19 was based on the confirmation of SARS-CoV-2 from RT-PCR using Roche COBAS Z480 analyzer (PT. Roche Indonesia, Jakarta). Severity classification was divided into 5 categories based on the Indonesia COVID-19 management guidelines, which are detailed as follows [15]:

(1) Asymptomatic case.
(2) Mild disease: symptomatic patients without evidence of viral pneumonia or hypoxia. Symptoms might appear as fever; cough; fatigue; anorexia; shortness of breath; myalgia; sore throat; nasal congestion; headache; diarrhea; nausea and vomiting; anosmia; or loss of appetite.
(3) Moderate disease: patients with clinical signs of pneumonia (fever, cough, shortness of breath, rapid breathing) but no symptoms of severe pneumonia, including oxygen saturation >93% on room air.
(4) Severe disease: patients with clinical signs of pneumonia (fever, cough, shortness of breath) plus respiratory rate >30 times/minute, severe respiratory distress, or SpO_2_ < 93% on room air.
(5) Critical disease: patients with acute respiratory distress syndrome (ARDS), sepsis, and septic shock.

### 2.2. Definition

The clinical parameters studied, such as age, gender, comorbidities (diabetes mellitus [DM], malignancy, autoimmune disease, human immunodeficiency virus [HIV] infection, liver cirrhosis, obesity), and symptoms, were retrieved from medical records. RT-PCR examination was conducted every five days based on the local hospital standard procedure. The duration of viral shedding was calculated from the onset of the symptoms until the day of 2 consecutive negative detections of SARS-CoV-2 RNA within 24 h [16,17,18]. The cut-off value for prolonged viral shedding was determined from the average duration obtained from data analysis. The duration of SARS-CoV-2 RNA shedding was considered prolonged if was is equal to or more than 13 days (Figure 1). The primer and probe for RT-PCR for SARS-CoV-2 detection targeted the N, E, and ORF1ab genes, with a cycle threshold (CT) value <38 considered positive.

### 2.3. Statistical Analysis

We analyzed the data using SPSS version 24 (Chicago, IL, USA; RRID: SCR_002865). The two groups were divided based on the average duration of viral shedding. The differences between the two groups were analyzed using the chi-square and Fisher’s exact tests because of the abnormally distributed data. The *p*-value that was considered significant was <0.05 with a confidence interval of 95%. Variables with significant differences in the bivariate analysis were included in the logistic regression multivariate analysis to determine factors that influenced the duration of SARS-CoV-2 viral shedding. The results were presented in the form of an odds ratio (OR) with the *p*-value considered significant being <0.05 and a confidence interval of 95%, and the adjusted OR (aOR) showed the results of the multivariate analysis.

### 2.4. Ethical Clearance

The ethical clearance (no. 0825/LOE/301.4.2/III/2022) for this study was granted by the Ethical Committee of Dr. Soetomo Hospital, Surabaya, Indonesia.

## 3. Results

### 3.1. Baseline Characteristics

The patient characteristics are shown in Table 1. This study included 162 patients as subjects with 83 (51.23%) men and 79 (48.76%) women. Most study participants (75.31%) were under 60 years old. The most frequent clinical symptoms were cough, fever, and dyspnea; some other symptoms were expectoration and hemoptysis. With respect to disease severity, mild cases were the most prevalent (43.83%) followed by severe cases (26.54%) and moderate cases (20.99%). The average length of hospitalization was 7.1 ± 8.82 days. Chest X-rays mostly showed bilateral pulmonary infiltrates (67.9%).

The most prevalent comorbidities were diabetes mellitus (33.95%) and hypertension (28.54%). Other comorbidities included malignancy, HIV/AIDS, liver cirrhosis, and autoimmune diseases. The antiviral treatments given were remdesivir (27.16%), favipiravir (22.84%), lopinavir/ritonavir (20.99%), and oseltamivir (15.43%). Oxygen therapy was delivered to 89 patients (54.94%).

Most patients were cured and discharged from the isolation ward (93.21%), while mortality was reported in 11 subjects (6.79%). The average length of stay was 7.41 ± 8.82 days. The median duration of viral shedding in this study was 10 days, with the shortest duration being 5 days and the most prolonged duration recorded being 42 days, with an interquartile range (IQR) of 7–17.25 days.

### 3.2. Bivariate Analysis

The bivariate analysis was based on the mean duration of viral shedding (Table 2). Most subjects aged <60, namely 79 subjects (79.8%), had viral shedding <13 days. Subjects aged 60 years and over had a more significant proportion who experienced prolonged viral shedding ≥13 days, although this difference was not statistically significant (*p* = 0.097). The duration of viral shedding was not significantly different for men and women (*p* = 0.816). However, there was a more significant proportion of more prolonged viral shedding for men (33 subjects; 52.4%) than for women. Patients with comorbid hypertension had a more considerable proportion who experienced a viral shedding duration of ≥13 days (38.1% vs. 22.2%), and the difference was statistically significant (*p* = 0.029). A total of 55 patients (33.95% of the total subjects) had DM, which was further categorized into DM with chronic complications in 12 patients (7.41% of the total subjects) and DM without chronic complications in 43 patients (26.54% of the total subjects). Most COVID-19 patients with DM had prolonged viral shedding (*p* = 0.001). The duration of viral shedding was not statistically different in COVID-19 patients with liver cirrhosis (*p* = 0.27). More COVID-19 patients with malignancy experienced viral shedding <13 days, although there was no statistically significant difference (*p* = 0.748). A total of 11 patients out of the total COVID-19 subjects (6.79%) experienced comorbid HIV/AIDS infection. Among these patients there was no significant difference in the duration of viral shedding with *p* = 0.27. Most of the COVID-19 patients with comorbid autoimmune diseases in this study had a course of viral shedding <13 days, but this difference was not statistically significant (*p* = 0.172). Obesity in this study was found in 9 patients (5.55%). There was no significant difference in the duration of viral shedding in obese patients with COVID-19 (*p* = 0.725). Patients who experienced dyspnea had a significantly longer duration of viral shedding (*p* = 0.011). Other symptoms, such as fever, cough, expectoration, and hemoptysis, did not differ substantially with respect to the duration of viral shedding.

### 3.3. Multivariate Analysis

The multivariate analysis of several variables with significant differences in the bivariate analysis included disease severity; dyspnea; bilateral pulmonary infiltrates on chest X-ray; hypertension; diabetes; and treatments including oxygen supplementation, antibiotics, oral antivirals, and corticosteroids. In the stepwise analysis, it was found that the factors related to the duration of viral shedding of SARS-CoV-2 were disease severity (aOR 2.94; CI 95%, 1.34–6.44), bilateral pulmonary infiltrates (aOR 2.79; CI 95%, 1.14–6.84), hypertension (OR 2.15; CI 95%, 1.08–4.32), and antibiotic therapy (aOR 3.66; CI 95%, 1.74–7.71). Age, gender, and symptoms were not associated with the duration of SARS-CoV-2 viral shedding (see Table 3).

## 4. Discussion

The characteristics of the research subjects revealed that gender had nearly equal proportions with 51.23% men and 48.76% women. Most research subjects (75.31%) were under 60 years old. This phenomenon might have been related to the disease severity pattern in this study, which was dominated by mild illness (43.83%). As reported in previous studies, older age is associated with COVID-19 mortality [19,20]. The elderly mostly exhibit severe or critical disease that requires ICU treatment and is not included in this study. The clinical symptoms observed in this study were similar to those reported by Qi et al., including cough, fever, and shortness of breath. Some of the patients also reported having expectoration and hemoptysis [16]. The most prevalent comorbidities reported in this study were diabetes mellitus and hypertension, followed by malignancy, HIV/AIDS infection, liver cirrhosis, and autoimmune diseases. Several studies have reported hypertension as the most prevalent comorbidity (11–83%), while in this study, hypertension was found in 28.4% of subjects [3,16,17,21].

This study’s reported mean duration of RNA viral shedding was 13 ± 8.44 days with a median of 10 days (interquartile range 7–17 days). Several similar studies have reported different mean and median viral shedding durations. A Chinese study in early 2020 involving 101 hospitalized patients reported a median viral shedding period of 11 days (interquartile range: 8–14.3 days) [3]. Another study of 147 patients at a COVID-19 treatment center outside Wuhan, China, reported a median viral shedding period of 17 days (interquartile range: 12–21 days) [16]. A multicenter retrospective cohort study during the early pandemic involving 191 inpatients reported a median viral shedding period of 20 days in survivors, but SARS-CoV-2 was detectable until death in non-survivors. The most prolonged observed duration of viral shedding in survivors was 37 days [19]. Differences in viral shedding times may have been due to a patient’s condition and disease severity. 

COVID-19 occurring in elderly patients is often associated with disease severity and the prolonged duration of SARS-CoV-2 viral shedding due to weakened immune systems and the presence of comorbidities. A study by Zhou et al. reported that older age was associated with the persistence of the SARS-CoV-2 virus; in these patients the virus could survive for 111 days in the respiratory tract [22]. This study found that more subjects over 60 experienced a longer duration of viral shedding ≥13 days, while subjects under 60 years experienced a shorter duration. However, the difference was not statistically significant. Several similar studies have also reported that there is no association between age and the duration of viral shedding [17,21]. This suggests that factors other than age contribute to the prolonged duration of viral shedding in SARS-CoV-2.

In this study, there was no difference in the viral shedding time of SARS-CoV-2 between men and women. This phenomenon has also been reported in several similar studies [16,18,21]. At the same time, a study by Xu et al. stated that the prolonged duration of SARS-CoV-2 viral shedding was found more commonly in men [17]. The widely reported influence of sex on COVID-19 is with respect to disease severity and mortality, where males tend to experience more severe cases with higher mortality. Immune responses and hormonal systems play a role in the pathogenesis of COVID-19, although this may still be multifactorial [23].

COVID-19 patients with hypertension had a significantly higher proportion who experienced a longer duration of viral shedding. A similar study by Xu et al. also reported prolonged viral shedding beyond 15 days in patients with comorbid hypertension [17]. Hypertension has been reported to be associated with COVID-19 disease progression and ARDS risk through oxidative stress mechanisms, endothelial dysfunction, and RAS modification [12,24].

Patients with type 2 diabetes mellitus are at risk of complications of COVID-19, and several researchers have reported an association between COVID-19 and diabetes. Type 2 diabetes mellitus is also a risk factor for prolonged viral shedding in SARS-CoV-2 [25]. In human monocytes, elevated blood sugar levels directly increase SARS-CoV-2 replication; glycolysis maintains SARS-CoV-2 replication through reactive oxygen species (ROS) production and the activation of hypoxia-inducible factor 1α; thus, hyperglycemia is a condition that supports viral proliferation [26]. In this study, DM patients experienced a significantly longer viral shedding time (*p* = 0.001). There was no significant difference in the duration of SARS-CoV-2 viral shedding in DM patients with chronic complications.

By contrast, there was a significant difference in the duration of viral shedding in uncomplicated DM. These results indicate that the influence of diabetes on the duration of viral shedding is determined by the diabetes status and not by the complications of diabetes. Some other studies have reported no significant difference in the course of viral shedding in COVID-19 patients with comorbid diabetes. All three of these studies mainly involved mild to moderate COVID-19 patients [3,16,17,21]. A study by Buetti reported that diabetes was a risk factor for the prolonged release of the SARS-CoV-2 virus, but the patients included in this study were COVID-19 patients in a critical condition [25].

The duration of viral shedding was not statistically different in COVID-19 patients with liver cirrhosis. Research on COVID-19 in the cirrhotic population is still very limited. Nonetheless, several other studies regarding the duration of viral shedding in COVID-19 patients with comorbid cirrhosis have reported no difference in the duration of viral shedding for SARS-CoV-2 [3,18,27].

The COVID-19 patients with malignancy in this study did not suffer differences in the duration of SARS-CoV-2 viral shedding. Another similar study also reported no difference in the period of viral shedding in COVID-19 patients with and without malignancy [3,9,18,21]. Some of these studies did not state the type of malignancy, such as hematological malignancies or solid tumors, as in this study. A case report of a COVID-19 patient with hematologic malignancy indicated a prolonged duration of viral shedding of up to 97 days [8].

This study showed no significant difference in the viral shedding time of COVID-19 patients with HIV/AIDS (*p* = 0.27) (Table 2). The depletion and impaired cellular function of CD4+ T cells might have been the cause behind the prolonged viral shedding time. The duration of SARS-CoV-2 viral shedding reported in HIV and COVID-19 coinfection was 28–85 days; it is therefore recommended that the isolation period of people living with HIV be longer than 14 days [28,29,30]. The different results of this study may have occurred due to the small sample of HIV/AIDS coinfection (6.79% of the total subjects). 

A total of 7 patients (4.32% of the total study subjects) had an autoimmune disease. In this study, most COVID-19 patients with an autoimmune disease had a viral shedding duration of <13 days, but the difference was not statistically significant, with *p* = 0.172 (Table 2). A disturbance of the immune system in autoimmune disease will affect the body’s ability to fight pathogens, including SARS-CoV-2. Treatment with immunosuppressants may also affect the prognosis of COVID-19 patients [31]. However, there have been no reports on the impact of autoimmune disease on the duration of SARS-CoV-2 viral shedding. 

In some literature reviews, obesity has been said to be associated with increased transmission rates, aggravating disease severity, increased mortality, and prolonged SARS-CoV-2 viral shedding [32,33]. A study by Zhang et al. reported that obesity was an independent risk factor for protracted SARS-CoV-2 viral shedding in the respiratory tract with an OR of 3.31; 95% CI: 1.08–10.09 [21]. The difference in the results of this study may have been due to the small number of obese patients included (5.55% of the total sample). The proportion of obese patients with a SARS-CoV-2 viral shedding duration ≥13 days was slightly higher than <13 days, but it was not statistically significant.

COVID-19 patients who presented with dyspnea in this study had a significantly longer duration of viral shedding. Dyspnea as the primary signal of worsening respiratory failure supported by the presence of lung lesions is common in moderate to severe COVID-19 cases. The extent of the opacity on the CT of the lungs is related to the duration of viral shedding in airway samples [34].

In influenza A virus infection, there is a relationship between tympanic temperature and viral shedding [35]. As the most common symptom of COVID-19 in this study, fever showed a trend related to the prolonged duration of SARS-CoV-2 viral shedding. However, this was not statistically significant, which was possibly due to the limited sample size. Another study reported that in addition to protracted viral shedding, higher temperatures were significantly associated with the progression of COVID-19. Thus, patients with high temperatures upon entry require more careful monitoring and the consideration of undergoing a more extended quarantine [16].

**Figure 2:**
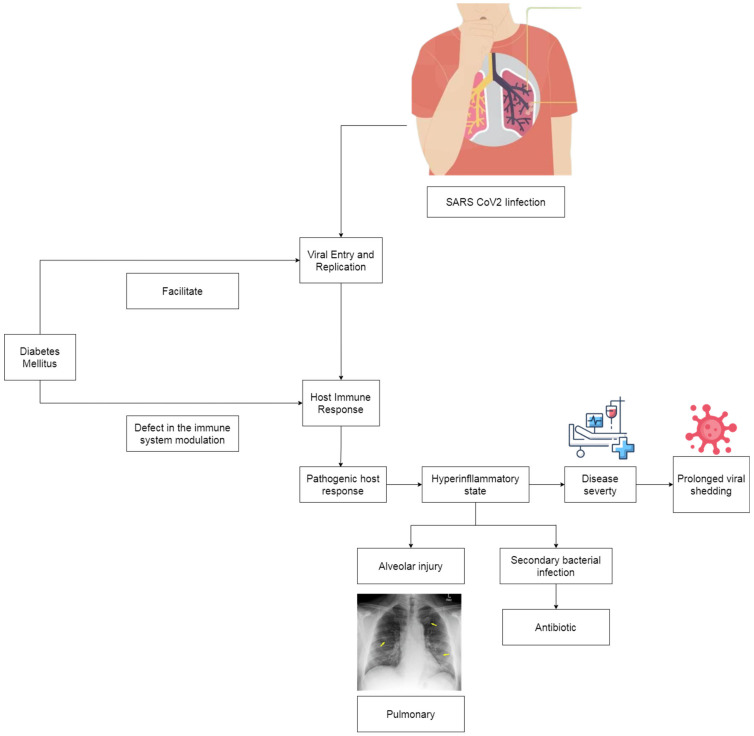
Factors associated with prolonged viral shedding in COVID-19 patients.

Based on this study, disease severity, bilateral pulmonary infiltrates on chest X-ray, and antibiotic therapy were independently associated with the duration of SARS-CoV-2 viral shedding. Figure 2 describes the proposed mechanism by which diabetes might prolong SARS-CoV-2 RNA shedding through viral entry and replication facilitation. Diabetes mellitus also affects the immune response to virus elimination. The pathogenic host response to SARS-CoV-2 could result in a hyperinflammatory state which determines disease severity. Disease severity related to a high viral load affects the length of viral clearance from the respiratory tract of COVID-19 patients [34]. The appearance of bilateral pulmonary infiltrates on a chest X-ray also reflects disease severity due to the excessive inflammatory response in COVID-19. Antibiotic therapy and its association with the duration of SARS-CoV-2 viral shedding may be related to bacterial coinfection commonly found in severe COVID-19 patients. Hence, it would affect the viral clearance of SARS-CoV-2. Previous studies have also reported that bacterial coinfection was more common in COVID-19 patients with poor outcomes [14,36].

This study’s limitation was that the viral shedding of SARS-CoV-2 was determined based on PCR viral particle detection; thus, the presence of viral shedding in the respiratory tract could not be defined. This research was conducted in June–December 2021 when the dominating SARS-CoV-2 variant was the Delta variant, which may have affected the severity and duration of SARS-CoV-2 shedding. Viral RNA sequencing tests are still not being conducted routinely. However, differences in the SARS-CoV-2 variants had minimal or no impact on the virus properties in general. The SARS-CoV-2 PCR tests in this study were carried out every five days based on the protocol applied in the hospital, thus allowing for bias in determining the duration of SARS-CoV-2 viral shedding. This study’s small sample size and observational method brought a high risk of confounding variables.

## 5. Conclusions

Several clinical parameters are related to the duration of SARS-CoV-2 RNA shedding. Disease severity is positively associated with the duration of viral shedding, while bilateral pulmonary infiltrates and antibiotic therapy are negatively related to the duration of viral shedding. Comorbidity diabetes mellitus is also associated with prolonged viral shedding. Overall, our findings suggest the need to consider different isolation period estimations for the specific clinical characteristics of patients with COVID-19 such as those with severe disease, bilateral pulmonary infiltrates, antibiotic treatment, and diabetes mellitus.

## Figures and Tables

**Table 1:** COVID-19 patient (cohort) characteristics.

Characteristics	Total (*n* = 162)
**Sex, *n* (%)**	
**Male**	83 (51.23)
**Female**	79 (48.76)
**Age, *n* (%)**	
**≥60 years**	40 (24.69)
**<60 years**	122 (75.31)
**Clinical symptoms, *n* (%)**	
**Fever**	83 (51.23)
**Cough**	85 (52.47)
**Expectorations**	35 (21.60)
**Hemoptysis**	1 (0.62)
**Dyspnea**	80 (49.38)
**Disease severity, *n* (%)**	
**Mild**	71 (43.83)
**Moderate**	34 (20.99)
**Severe**	43 (26.54)
**Critical**	14 (8.64)
**Length of hospitalization, mean ± SD**	7.41 ± 8.82
**Chest X-ray, *n* (%)**	
**Normal**	32 (19.75)
**Unilateral infiltrate**	15 (9.26)
**Bilateral infiltrates**	110 (67.90)
**Atypical**	5 (3.09)
**Comorbidities, *n* (%)**	
**Diabetes mellitus**	55 (33.95)
**DM with chronic complication**	12 (7.41)
**DM without chronic complication**	43 (26.54)
**Hypertension**	46 (28.40)
**HIV/AIDS**	11 (6.79)
**Malignancy**	17 (10.49)
**Liver cirrhosis**	11 (6.79)
**Autoimmune disease**	7 (4.32)
**Obesity**	9 (5.55)
**Treatment, *n* (%)**	
**Antiviral therapy:**	
**Lopinavir/ritonavir**	34 (20.99)
**Favipiravir**	37 (22.84)
**Remdesivir**	44 (27.16)
**Oseltamivir**	25 (15.43)
**Corticosteroid**	63 (38.89)
**Convalescent plasma**	6 (3.70)
**Antibiotic**	77 (47.53)
**Oxygen therapy**	89 (54.94)
**Output, *n* (%)**	
**Death**	11 (6.79)
**Cured**	151 (93.21)
**Duration of viral shedding (days) (Mean ± SD)**	13 ± 8.44

**Table 2:** Characteristics based on the duration of viral shedding.

Characteristics	Total (*n* = 162)	Duration of Viral Shedding		*p*-Value
		<13 days (*n* = 99)	≥13 days (*n* = 63)	
Sex				
Male	83	50 (50.5%)	33 (52.4%)	0.816
Female	79	49 (49.5%)	30 (47.6%)	
Age				
≥60 years	40	20 (20.2%)	20 (31.7%)	0.097 *
<60 years	122	79 (79.8%)	43 (68.3%)	
Clinical symptoms				
Fever	83	45 (45.5%)	38 (60.3%)	0.065 *
Cough	85	48 (48.5%)	37 (58.7%)	0.203 *
Expectoration	35	18 (18.2%)	17 (27.0%)	0.184 *
Hemoptysis	1	1 (1.0%)	0 (0%)	0.484
Dyspnea	80	41 (41.4%)	39 (61.9%)	0.011 *
Disease severity				
Mild	71	50 (50.5%)	21 (33.3%)	0.012 *
Moderate	34	24 (24.2%)	10 (15.9%)	
Severe	43	19 (19.2%)	24 (38.1%)	
Critically ill	14	6 (6.1%)	8 (12.7%)	
Chest X-ray				
No infiltrate	32	26 (26.3%)	6 (9.5%)	0.009 *
Unilateral infiltrate	15	12 (12.1%)	3 (4.8%)	0.115 *
Bilateral infiltrate	110	56 (56.6%)	54 (85.7%)	0.00 *
Atypical	5	4 (4.0%)	1 (1.6%)	0.379
Comorbidities				
Diabetes Mellitus	55	24 (24.2%)	31 (49.2%)	0.001 *
- with chronic complications	12	5 (6.1%)	7 (9.5%)	0.151 *
- without chronic complications	43	18 (18.2%)	25 (39.7%)	0.003 *
Hypertension	46	22 (22.2%)	24 (38.1%)	0.029 *
HIV/AIDS	11	5 (5.1%)	6 (9.5%)	0.27
Malignancy	17	11 (11.1%)	6 (9.5%)	0.748
Liver cirrhosis	11	5 (5.1%)	6 (9.5%)	0.27
Autoimmune disease	7	6 (6.1%)	1 (1.6%)	0.172 *
Obesity	9	5 (5.1%)	4 (6.3%)	0.725
Treatment				
Lopinavir/ritonavir	34	10 (10.1%)	24 (38.1%)	0.00 *
Favipiravir	37	29 (29.3%)	8 (12.7%)	0.014 *
Remdesivir	44	27 (27.3%)	17 (27.0%)	0.968
Oseltamivir	25	17 (17.2%)	8 (12.7%)	0.442
Corticosteroid	63	32 (32.3%)	31 (49.2%)	0.032
Convalescent plasma	6	2 (2.0%)	4 (6.3%)	0.155 *
Antibiotic	77	36 (36.4%)	41 (65.1%)	0.00 *
Oxygen therapy	89	44 (44.4%)	45 (71.4%)	0.001 *
Outcome				
Death	12	7 (7.1%)	4 (6.3%)	0.859
Cured	151	92 (92.9%)	59 (93.7%)	

* *p*-value chi-square and Fischer exact test <0.25.

**Table 3:** Multivariate analysis of parameters potentially associated with SARS-CoV-2 viral shedding.

Variable	Multivariate Analysis			Stepwise Analysis			
	OR	95% CI	*p*-Value	aOR	95% CI	*p*-Value	
≥60 years	1.88	0.89–3.78	0.09				
Disease severity	3.05	1.56–5.97	0.001 *	2.94	1.34–6.44	0.007 *	
Fever	1.82	0.96–3.46	0.065				
Dyspnea	2.29	1.20–4.39	0.011 *				
Cough	1.51	0.79–2.86	0.203				
Expectoration	1.66	0.78–3.54	0.184				
No infiltrate	0.299	0.11–0.76	0.009 *				
Unilateral infiltrate	0.363	0.09–1.34	0.115				
Bilateral pulmonary infiltrates	4.61	2.05–10.35	0.000 *	2.79	1.14–6.84	0.025 *	
Hypertension	2.15	1.08–4.32	0.029 *				
Diabetes mellitus	3.027	1.54–5.94	0.001 *	2.17	1.02–4.63	0.046 *	
Autoimmune disease	0.25	0.03–2.13	0.172				
Oxygen therapy	3.13	1.59–6.14	0.001 *				
Antibiotic	3.26	1.69–6.31	0.000 *	3.66	1.74–7.71	0.001 *	
Oral antiviral	1.36	0.70–2.62	0.36	1.96	0.89–4.29	0.093	
Corticosteroid	2.03	1.06–3.88	0.032 *				

OR, odds ratio; aOR, adjusted odds ratio. * *p*-value < 0.05.

## Data Availability

The raw data are available in DOI: https://doi.org/10.6084/m9.figshare.22724183.
